# Review of HIV Testing Efforts in Historically Black Churches

**DOI:** 10.3390/ijerph120606016

**Published:** 2015-05-28

**Authors:** Latrice Crystal Pichon, Terrinieka Williams Powell

**Affiliations:** 1 School of Public Health, University of Memphis, 209 Robison Hall, Memphis, TN 38152, USA; 2 Bloomberg School of Public Health, Johns Hopkins University, 615 N. Wolfe Street, E4614 Baltimore, MD 21205, USA; E-Mail: twilliams@jhu.edu

**Keywords:** HIV testing, church, religion, Black, African American

## Abstract

This paper aims to critically assess the state of HIV testing in African American churches. A comprehensive review of peer-reviewed publications on HIV testing in church-based settings was conducted by two independent coders. Twenty-six papers published between 1991 and 2015, representing 24 unique projects, were identified addressing at least one dimension of HIV testing. Thirteen faith-based projects have implemented HIV testing events or had clergy promote the importance of testing and knowing one’s HIV status, but empirical data and rigorous study designs were limited. Only eight papers reported onsite HIV testing in churches. Less than 5% of the studies reported the percentage of congregants who returned for their test results. Finally, no study has examined at baseline or post-intervention behavioral intentions to be screened for HIV. Future research is needed to evaluate the effectiveness of HIV testing in churches and to explore the possibilities of the role of the church and leadership structure in the promotion of HIV treatment and care.

## 1. Introduction

The Centers for Disease Control and Prevention (CDC) estimates that 16% of U.S adults and adolescents infected with HIV are unaware of their HIV-positive status [1]. Among Black Americans, the number is disproportionately greater; in 2010, almost 85,000 Black Americans infected with HIV were unaware of their HIV status [2]. An untreated or undiagnosed condition can lead to a number of consequences such as a compromised immune system and HIV transmission to sexual partners [3,4]. One of the most cost effective and efficacious ways to screen for HIV is through community-based HIV testing efforts and linkage to care [5].

The U.S. Preventive Services Task Force (USPSTF) recommends HIV testing for people 15–65 years old as a routine part of healthcare [6]. Researchers estimate that routine testing would facilitate early HIV diagnosis and likely lead to a 30% reduction in HIV incidence rates [7]. In 2011, there were over three million CDC-funded testing events. The largest proportion was conducted in healthcare and correctional facilities and in Black communities. A smaller proportion have been conducted in non-health care or non-traditional settings like churches [8]. In recognition of the National HIV/AIDS Strategy (NHAS) which calls upon all sectors, including the faith community, to work toward reducing HIV incidence, increasing access to care and improving health outcomes, and reducing HIV disparities, church settings offer a broader reach to achieve these goals.

Research from the Pew Forum on Religion and Public Life indicates that 85% of Black Americans report religion as being very important to them and more than half of Black Americans report attending religious services at least once a week [9]. Congregations include individuals who would benefit from increased HIV testing efforts who would otherwise not have access to testing or may forego testing because they do not perceive themselves to be at risk for HIV [10]. Furthermore, Black churches typically serve a wide age range of men and women with varying levels of HIV risk [11,12]. For instance, female congregants (n = 142) were surveyed from 14 churches in the North Carolina and 84% of the women reported a history of sexually transmitted infections [10]. Given these data, there is a need to explore how churches can be an access point for HIV testing and a possible linkage to HIV healthcare and services for Black communities. This may be the logical next step given the Black Church community has been involved in HIV prevention efforts since the onset of the epidemic nearly three decades ago [13]. Thus, in this paper we take a closer review of HIV testing in Black churches in the context of the HIV prevention continuum of care.

## 2. Methods

The literature reviewed in the present paper was identified from health and social science electronic databases including PubMed, PsychInfo, JSTOR, ATLA, and Scopus. MeSH and key search words included: “Black Church”, “HIV”, “clergy”, and “faith-based”. We included papers published between June 1, 1991 to April 20, 2015 meeting the following conditions: (1) addressed HIV testing among U.S. Black religious congregations, (2) described the recruitment method, design, and/or data obtained. Reference sections from papers retrieved were examined to locate additional articles. 

A total of 221 unduplicated papers were initially reviewed. Papers that did not describe the recruitment method, design, and/or include data were excluded. Seventy-five papers remained. Next, we identified those papers addressing HIV testing. We independently reviewed each paper and met weekly to discuss discrepancies until consensus was reached. Based on the consensus between the research team, 26 papers representing 24 unique projects were reviewed (Table 1).

## 3. Results

Church-based HIV testing events were typically held in partnership with outside health agencies (e.g., health departments, CDC, and community-based organizations with an HIV focus). The number of churches with programs or interventions varied across studies, but ranged from 4 to 120. Although engaged in a range of HIV testing efforts, none of the church-based programs reviewed reported HIV testing as a main outcome. Only eight papers reported onsite HIV testing in churches. Of those eight papers, the number of congregants tested was low or not reported. Moreover, less than 5% of the studies reported the percentage of congregants who returned for their test results. Finally, no study has looked at baseline or post-intervention behavioral intentions to be screened for HIV among church members participating in a faith-based HIV intervention or program.

Berkley-Patton and colleagues were the first to report on HIV testing behaviors of a church-based sample (n = 210) participating in a faith-based HIV awareness and testing intervention [14]. Fifty-six percent of church members felt more strongly encouraged to get an HIV test by their fellow church members than friends or family [15]. Predictors of HIV testing among the church sample included not being married, not having insurance, inconsistent condom use, and intentions to test in the future [15].

One study demonstrated faith leaders’ ability to promote HIV testing through premarital counseling, reduction of stigma, and normalizing screening behaviors. Aholou *et al.* found pastors encouraged couples to test for ‘peace of mind’ [16]. Other papers have shown church leaders stress the importance of knowing one’s status [17] and HIV testing is perceived by church leaders as a way to reduce HIV stigma and support those infected and affected by HIV [18]. Faith leaders also recommended the importance of clergy undergoing testing [19,20] and promoting routine testing to congregations from the pulpit [21]. Outcomes typically reported in these cases are the number of individuals screened at churches [18,19,22,23]. There were only two papers that reported data on the receipt of HIV test results at church-based testing venues. For both rapid and conventional HIV testing, Aaron *et al.* reported 100% return rates among church congregants [18]. Whiters *et al.* identified a total of 85 HIV-infected individuals from a larger sample of 1947 persons tested for HIV from January 2004 to July 2005 in an HIV testing initiative of a coalition of six Black churches [24].

**Table 1:** HIV testing program/study characteristics.

First Author (Year)	Type of Data Presented	Summary of HIV Testing Efforts
Aaron (2011) [18]	Description/Development—coalition (program)	Congregation identified HIV testing as a need; 145 parishioners screened for HIV
Agate (2005) [22]	Mixed methods (program)	Pastors from 120 churches formed consortium providing HIV testing to over 825 people
Aholou (2011) [16]	Qualitative—interviews (study)	Pastors encouraged couples to get HIV test for “peace of mind”
Alder (2007) [25]	Qualitative—focus groups (study)	Eleven clergy indicated a sense of responsibility to address HIV testing and care within their theological framework and spheres of influence
Berkley-Patton (2012) [14]	Quantitative—surveys (study)	87% of congregants believed it was important for their church to talk about HIV testing
Cunningham (2009) [26]	Qualitative—interviews	Five of 16 churches provided HIV testing on-site during the previous year
Francis (2009) [27]	Quantitative—mailed surveys	63% willing to provide or offer HIV testing at their church
Griffith (2010) [19]	Description/Development—training	n = 80 tested for HIV at pilot study events and pastors were often the first ones in line; expansion project tested n ≥ 400 for HIV at faith-based events and 4 churches served as testing sites;
Hicks (2005) [20]	Qualitative—interviews	Two of 9 church-or FBO case examples offered HIV testing
Khosravani (2008) [28]	Quantitative—surveys	18.5% (n = 59 of 319) of congregation members stated their church provided HIV testing; 4 of 12 ministers arranged for free HIV testing for their members
Kruger (2010) [23]	Quantitative—survey interviews	26% (total sample n = 97) from FBOs tested for HIV in the last year
Lindley (2010) [29]	Quantitative—surveys	29.5% of parishioners and 20.3% of pastors responded incorrectly to the statements that it was possible, but unlikely, to get HIV from an HIV test
MacMaster (2007) [30]	Description—testing services	Program goal was to provide HIV/STD testing and counseling services for substance users
Moore, D.D. (2010) [31]	Qualitative—interviews	Implemented HIV testing at 18 churches; 229 of 798 got tested
Moore, D (2012) [17]	Qualitative—interviews	Encouraged and provided HIV testing onsite (invited health dept. back for more tests)
Nunn (2012) [21]	Qualitative—multiple methods	Faith leaders suggested pastors should promote routine HIV testing and undergo HIV testing to destigmatize and encourage widespread testing
Palar (2012) [32]	Qualitative—case study	Seven out of 14 congregations have done HIV testing
Pichon (2012) [33]	Quantitative—survey interview	Salient quote—Chairperson of Worship “HIV testing was helpful. All the kids got tested”
Smith (2005) [34]	Quantitative—surveys	A list of the types of HIV programs offered emphasized 0 referrals for HIV testing
Sommerville (2008) [35]	Description—case study	Offers solutions: (1) Advocate for policies that ensure and expand HIV testing, and (2) Create safe places in churches where persons can be confidentially and regularly tested
Stewart (2012) [36]	Qualitative—ethnographic case study	One of 5 intervention sessions covered HIV testing
Tesoriero (2000) [37]	Quantitative—surveys	Less commonly offered services included referrals for HIV testing
Werber (2012) [38]	Qualitative—case study	Salient participant quotes about HIV testing
Whiters (2010) [24]	Quantitative—case study—	Collaboration with churches to implement rapid HIV testing to 1947 substance users
Wingood (2011) [39]	Description/Development—intervention	Intervention session designed to increase participants’ awareness of getting oneself and one’s partner tested
Wooster (2011) [40]	Qualitative—interviews	Participants indicated more HIV testing were available within churches than before; benefit to providing HIV testing services at a church was to seek spiritual support while getting tested; Participants attributed the increase in church requests for onsite testing services to an HIV testing campaign

## 4. Discussion

Offering HIV testing in Black churches has increased in acceptability as churches have opened their doors to external agencies to bring HIV testing to their congregations [17,36,41]. Today, churches often collaborate with certified HIV testers to conduct on-site testing as members of their health ministry are not always equipped with this skillset [32,38,42]. There are time constraints and infrastructure challenges for church leaders to become certified [20]. Churches find it more feasible to work with testing agencies as a collaborative as opposed to being a certified testing site [20]. These types of partnerships also have promise when working with high-risk vulnerable populations, and dealing with sensitive issues such as substance use and sexuality as external agencies have experience and the expertise to handle these matters [24].

Church-based testing is aligned with the anticipated results of the National HIV/AIDS Strategy to increase the percentage of people who know their serostatus. The current review adds to the scientific base/knowledge a comprehensive/critical review of the current state of published work around HIV testing in churches. Previous reviews examined HIV health education programs and interventions in faith-based organizations. We are moving away from primary prevention and are using test-and-treat models. Thus, this paper provides a different lens by demonstrating the lack of connection between efforts and: (1) routine testing, (2) reduction in HIV incidence, and (3) linkage to care for positives. Additionally, it highlights the underutilization of collaborations between faith-based organizations and public health for HIV testing. Other reviews have focused on the formation of collaborative research partnerships with faith leaders and academics. While this is crucial, it is suggested future studies include the evaluation of such CBPR partnerships as it relates to HIV testing. We need to document the strategies and methods used to assess HIV testing interventions in churches. Furthermore, it is important to document the change in HIV testing norms experienced by churches.

### 4.1. Strengths and Limitations of Church-Based HIV Testing Research

This study is not without limitations. First, faith-based HIV research is in its infancy. We chose to focus the analysis only on peer-reviewed published work included during the last 24 years as the numbers of peer-reviewed publications have grown. We purposely did not review the grey literature (no book chapters, editorials, commentaries) as the peer-review process is the gold standard. It was important to build a case for the growing need to increase the rigor of this research. Thus, our results/papers reviewed may have differed in the absence of these limitations. Future recommendations include conducting a grey literature search of dissertations, editorials, and op ed pieces once the science has been established. The utility of the grey literature has merit/value but given the infancy of this research and the scope of this paper we thought it best to streamline our efforts. Second, there is a dearth of pre/posttest research designs that evaluate the effectiveness of the promotion of church-based HIV testing programs on congregational testing behaviors. Developing strategies to assess the rate of HIV positives that are identified at church-based functions is needed to support the continued need for these programs. Furthermore, there is a gap in surveillance data showing the rate of HIV positives that are identified during church-based testing events.

Despite these limitations, there are several strengths to this study. First, this paper documents the scope of HIV testing events in churches. There is a growing body of literature documenting HIV testing events in churches as many reported testing hundreds of members at a given time [14,44]. Second, our findings suggest that many pastors are strong supporters of knowing your status and promoting HIV testing. Third, our findings suggest that congregational members return for test results more frequently when tested in churches than when tested in other community settings [43]. However, very few churches offer testing referral services [28,34,37]. Furthermore most church-based HIV testing activities have not been rigorously studied or evaluated and have mostly been exploratory with the exception of one promising research study. 

### 4.2. Public Health Implications 

This paper adds to scientific knowledge a critical review of the current state of published work around HIV testing in Black churches. Somerville offers solutions for clergy to be advocates for testing policy changes and to create spaces in churches for congregations and other community members to receive confidential testing [35]. We encourage the field to extend this by providing more support for the development of evidence-based HIV testing programs in church communities. A logical next step for future research would be to evaluate the effectiveness of HIV testing in churches and the rate of identified HIV positive participants yielded from these efforts. However, better study designs and measurements of HIV testing behaviors of congregations are needed. In particular, it would be important to design and conduct a longitudinal study to follow-up with church leaders to understand how and if at all HIV testing had been institutionalized in the church. 

There is a definite need to collaborate across different sectors for implementing HIV testing efforts in churches. Without the collaborative spirit and skill-set of all partners involved the mere success of executing HIV testing in churches would not be possible. Normalizing HIV testing in faith contexts can be achieved with the assistance of church leadership. Additionally, linking church and community members to HIV care and support services should be explored particularly among younger age groups. However, to do this, the issue of privacy and HIV stigma reduction would be necessary to address. Nevertheless, the current numbers of individuals linked to care are well below NHAS objectives. To this end, future work needs to explore the possibilities of the role of the church and leadership structure in the promotion of HIV treatment and care and medication adherence with particular emphasis on youth and young adults as the promotion of testing were not always geared toward this population. A glaring gap is the lack of involvement of young people in the research process and stakeholders from both secular and non-secular spheres in the conceptualization of culturally appropriate HIV testing interventions for at-risk priority groups.

## 5. Conclusions 

Historically Black Churches have been involved in HIV prevention efforts since the onset of the epidemic three decades ago. Although the scope and breadth of education, awareness, and outreach efforts vary, there is evidence that many Black churches are engaged in HIV prevention efforts today. Twenty-four U.S. faith-based HIV prevention projects reported findings from church-based testing events, church-wide surveys on testing uptake, and congregational members’ perceptions of availability of testing. Furthermore, evidence suggests faith leaders who receive HIV training tools are more comfortable counseling HIV positive adults, delivering HIV-related sermons, sponsoring training sessions in their church, and hosting programs at their church. The findings and recommendations from the current study should encourage and aid future researchers in their efforts to work with Black Churches not only on HIV prevention, but also across the HIV continuum of care.
